# Concurrent infections by *Bonamia* species (Haplosporidia) do not cause more intense infections

**DOI:** 10.1017/S0031182025100978

**Published:** 2026-01

**Authors:** Henry S. Lane, Jaret Bilewitch, Amber Brooks, Lisa Smith, Marine Pomarède, Megan Dymond, Keith Michael, Felix Zareie-Vaux

**Affiliations:** 1 National Institute of Water and Atmospheric Research Ltd, Coasts and Estuaries, Wellington, New Zealand; 2 Fisheries New Zealand, Ministry for Primary Industries, Wellington, New Zealand; 3 Biosecurity New Zealand, Ministry for Primary Industries, Wellington, New Zealand

**Keywords:** co-evolution, coinfection, ddPCR, invasive species, marine disease, microcell parasite

## Abstract

Recently introduced parasites are predicted to cause more severe infections because of a lack of host-parasite co-evolution. When new parasites co-occur with similar parasites they may compete for resources within a host, with mixed species infections potentially resulting in antagonistic, synergistic or additive effects. We tested *Ostrea chilensis* flat oysters in New Zealand for infections by two species of haplosporidian oyster parasites. *Bonamia exitiosa* is an endemic parasite to New Zealand, whereas *Bonamia ostreae* is an introduced species first detected in New Zealand in 2015. We investigated the infection intensity of each parasite by estimating gene copy numbers using species-specific digital droplet PCR (ddPCR) across *Bonamia* spp. allopatric and sympatric ranges. Our results showed that *B. ostreae* had significantly higher gene copy numbers than *B. exitiosa*. However, concurrent infections of both *Bonamia* parasites had similar intensities (based on gene copy number) to single-species infections, with no detectable interactive effects. Collectively, the results indicate that *B. ostreae* remains a significant risk to *O. chilensis*, although coinfections may not exacerbate disease. This study demonstrates the value of ddPCR screening and the importance of considering evolutionary ecology in the management of commercially important marine diseases.

## Graphical abstract

**Figure:**
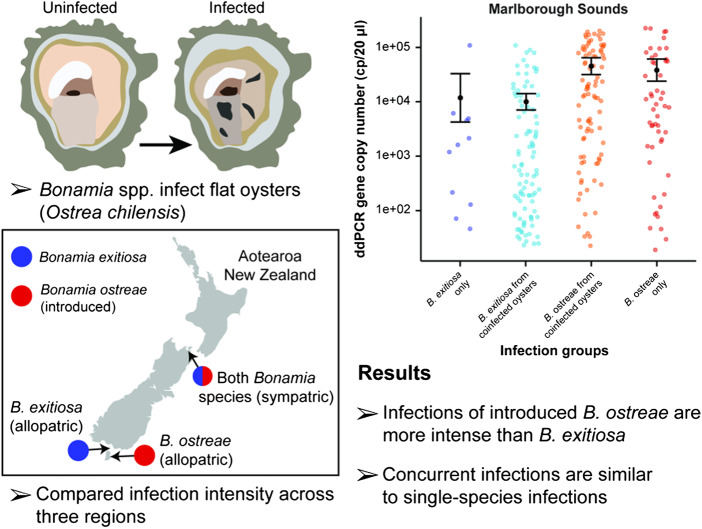

## Introduction

Invasive parasites pose a significant threat to new hosts and ecosystems, often causing elevated mortality and altered disease dynamics in their hosts due to lack of coevolution and mutual adaptation (Burreson et al. (2000); Dunn 2009; Poulin 2017; Poulin et al. 2011). Many introductions of marine parasites have caused widespread disease and high mortality in immunologically naïve host populations (Burreson et al. (2000); Corbeil 2020; Friedman et al. 2000; Keeling et al. 2014; Whittington et al. 2008). The introduction of a new parasite is also expected to affect other parasites, especially those with similar life histories. The nature and long-term impacts of inter-parasite interactions, and their effects on hosts, is largely unknown but important for understanding marine disease dynamics.

**Figure 1.**
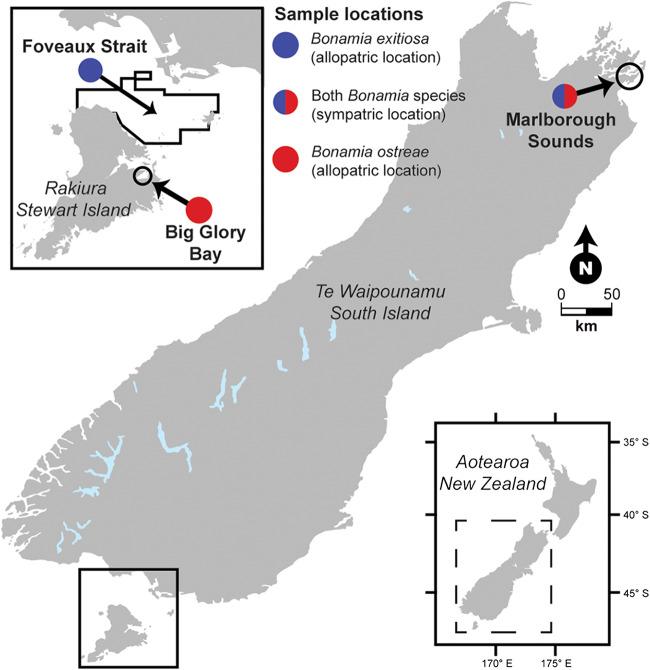
Illustrated map showing the confirmed locations for *Bonamia exitiosa* and *Bonamia ostreae* in New Zealand. Black lines show the approximate spread of collection sites within each sample location. See Table 1 for further information regarding the sample locations.

Two pathogenic haplosporidian oyster parasites *Bonamia exitiosa* and *Bonamia ostreae* infect New Zealand flat oysters *Ostrea chilensis* (Dinamani et al. 1987; Lane et al. 2016). *Bonamia exitiosa* has a long history in New Zealand and is known to infect *O. chilensis* across much of their range, from Hauraki Gulf in the north to Port Adventure in the deep south (Dinamani et al. 1987; Hine et al. 2001; Lane et al. 2018). *Bonamia ostreae* was first detected in New Zealand in 2015 (Lane et al. 2016) and has since been confirmed in *O. chilensis* in the Marlborough Sounds and in Big Glory Bay, Stewart Island (Figure 1) (Lane et al. 2016; NIWA 2021). The detection of *B. ostreae* in New Zealand revealed *O. chilensis* coinfected with *B. exitiosa* (Lane et al. 2016). Concurrent *Bonamia* sp. infections have also been reported in *Ostrea edulis* from Italy, Spain and England, but with no data presented beyond detection prevalence (Abollo et al. 2008; Narcisi et al. 2010; Longshaw et al. 2013; Lane et al. 2016).

*Bonamia exitiosa* and *B. ostreae* have direct intra-haemocytic lifecycles. Individual parasites infect and replicate within phagocytic haemocytes, leading to parasite proliferation and host death (Hine and Wesney 1994; Hine 1996; Hine et al. 2001, 2014; Comesaña et al. 2012). Given their evolutionary relatedness and similar life histories, sympatric *B. exitiosa* and *B. ostreae* could interact and compete for resources – namely the host haemocytes used for survival and reproduction. In situ hybridization (ISH) of concurrently infected animals has revealed that both parasites can be adjacent to one another in host tissues (Lane et al. 2016). Mixed infections of congeneric pathogenic parasites, such as vector borne terrestrial diseases like malaria and leishmania in vertebrates, can present more severe disease symptoms than single species infections (de Lima Celeste et al. 2017; Shen et al. 2019; Kotepui et al. 2020; Tang et al. 2020). Mixed infections can also have varied antagonistic, additive, and synergistic effects, altering virulence and influencing host phenotype and reproductive fitness (Hafer and Milinski 2015; Bose et al. 2016; Bolnick et al. 2024). Understanding disease dynamics within the oyster-*Bonamia* system is important for managing long-term impacts on host populations and oyster production. In New Zealand, bonamiasis has historically been caused by *B. exitiosa*, but the effects of *B. ostreae*, alone or in coinfection with *B. exitiosa*, remain unclear.

Droplet digital PCR (ddPCR) is a molecular testing technology similar to quantitative PCR (qPCR) that amplifies target DNA using thermocycling, but it partitions samples into thousands of droplets, enabling absolute quantification (Hindson et al. 2011; Pinheiro et al. 2012). This process removes the need for standard curves to provide a measurement of ‘gene copy number,’ which reflects how often a target sequence is detected. Gene copy number can serve as a proxy for infection intensity, with higher values indicating more intense infections (and vice versa; Bilewitch et al. 2018; Barrett-Manako et al. 2021; Howells et al. 2021). Species specific ddPCR assays have been designed and validated for *B. exitiosa* and *B. ostreae* in *O. chilensis* (Bilewitch et al. 2025). During assay validation, the number of *B. exitiosa* 18S rRNA gene copies detected was positively correlated with the number of *Bonamia* cells observed by cytology. Gene copy numbers increased with infection grade, with oysters classified as very heavy (grade 5) showing higher copy numbers than those with moderate (grade 3) or light infections (grade 1) (Bilewitch et al. 2018). A similar relationship was observed during diagnostic validation comparing detection of *B. ostreae* using histopathology and ddPCR (Bilewitch et al. 2025). Although specific quantitative test comparisons have only been carried out for *B. exitiosa,* we expect a similar parasite-gene copy number relationship for *B. ostreae* given their similar biology.

We used the two species-specific *Bonamia* ddPCR assays to investigate how infection intensities, inferred by gene copies, vary between an endemic and introduced parasite across allopatric and sympatric locations. We hypothesised that recently introduced *B. ostreae* would produce heavier infections, with higher gene copy numbers, than *B. exitiosa*, which has coevolved with their flat oyster hosts for longer. We expected that, given their direct intra-haemocytic life cycles, coinfection by the two parasite species would increase haemocyte parasitism and replication, resulting in higher gene copy numbers than in single species infections.

## Materials and methods

### Oyster samples

We used three pre-existing flat oyster collections from ongoing *Bonamia* spp. surveillance (Figure 1), which represent one sympatric and two allopatric sample locations for the *Bonamia* parasites: (1) oysters collected from the Marlborough Sounds in the upper South Island where *B. exitiosa* and *B. ostreae* are sympatric; (2) oysters collected from Big Glory Bay, Stewart Island where *B. ostreae* has been present since 2017 and there are no reported detections of *B. exitiosa*; and (3) oysters collected from Foveaux Strait, which remains free of *B. ostreae*, but where *B. exitiosa* was first reported in 1985 (Dinamani et al. 1987) and later identified in archived specimens from the 1960s (Hine and Jones 1994) (Figure 1). The prevalence of *B. exitiosa* in Marlborough Sounds was around 3%, *B. ostreae* was around 40% and concurrent infections around 50% (Lane et al. 2016). *Bonamia ostreae* has been detected at increasing prevalence in Big Glory Bay since its detection and is now around 30% (Bonamia Programme team 2023). Detection prevalence for *B. exitiosa* in Foveaux Strait sits at around 20% across the fishery area (Michael et al. 2023b).

Oysters from the Marlborough Sounds were collected in November 2014 and February 2015 from two farmed populations (Lane 2018). Wild oysters from Big Glory Bay were collected during the six-monthly austral Spring and Autumn *B. ostreae* surveillance sampling conducted between 2021 and 2024 (NIWA 2021; Bonamia Programme team 2023; Bolnick et al. 2024). Wild oysters from the Foveaux Strait were collected during the annual late austral Summer/early Autumn *B. exitiosa* surveillance between 2021 and 2024 (Michael et al. 2022, 2023a, 2023a). The Foveaux Strait oysters were collected from 12 fixed target stations (see T-prefix in Michael et al. 2022). These fixed target stations were selected because they sample the same geographic location every year, which establishes a time series of data from across the entire Foveaux Strait flat oyster fishery. All sampled oysters were sexually mature. Marlborough Sounds and Foveaux Strait have marine salinities (∼35 psu), while Big Glory Bay has a salinity of around 18-20 psu.

### ddPCR method

We screened heart tissues from all oysters using the species-specific *Bonami*a ddPCR assays that target the nuclear ribosomal 18S rRNA gene (Bilewitch et al. 2025). A dual-probe ddPCR assay targeted the 18S rRNA gene of the parasite and the oyster β-actin gene (which served as an internal positive control for DNA extraction and amplification of each sample). Each ddPCR reaction was 24 µL in volume and consisted of BioRad ddPCR SuperMix, primers and probes, and 3 µL of the diluted tissue digest. A QX200 AutoDG Droplet Digital PCR System (Bio-Rad, Hercules, CA, USA) was used to automate droplet generation prior to amplification on a CFX PCR thermocycler (Bio-Rad, Hercules, CA, USA) and droplet reading on a QX200 Droplet Reader (Bio-Rad, Hercules, CA, USA). All plates were run with two additional controls: a synthetic positive control standard and a negative template control.

All heart tissue specimens, or heart tissue digests, were frozen in 96-well PCR plates archived at NIWA, Wellington on behalf of Biosecurity New Zealand and Fisheries New Zealand. For consistency, we tested heart tissue only across samples because gills may have surface contamination from environmental parasites and may not accurately represent an infection.

Only samples displaying successful amplification of the oyster β-actin internal control were included. Test outliers with no negative droplets (i.e. the samples are completely saturated with positive droplets) were excluded from subsequent analyses since Poisson calculations of target concentration were not possible. All test ddPCR results were converted to number of copies per 20 µL (cp/20 µL) and were tabulated in Microsoft Excel.

### Statistics

A negative binomial generalized linear model (GLM) was used to test for differences in 18S rRNA gene copy numbers of *Bonamia* species among infection groups, defined by single versus concurrent infections across sympatric and allopatric sites. Marginal plots were generated to visualise estimated mean gene copies for each group and their 95% confidence intervals. A negative binomial GLM was chosen to account for overdispersion in the data (θ ranged from 0·27 to 0·34 across the four models [parasite sympatry in Marlborough Sounds, *B. exitiosa*-only, *B. ostreae*-only, and all location and infections groups]). Cook’s distance was calculated to identify potentially influential data points. All statistical analyses were performed using the R Statistical Software (v.4.4.2; R Core Team 2024) with the MASS package (v. 7.3.60.0.1) for fitting negative binomial GLMs, the emmeans package (v. 1.11.2) for estimated marginal means, and ggplot2 (v. 3.5.2) for visualization.

## Results

A total of 409 ddPCR results were analysed (Table 1). Our sampling included single-species infections (*B. exitiosa* or *B. ostreae*), as well as coinfections where oysters were infected simultaneously by both parasites. Sampling was approximately even across infection groups (i.e. single infections and coinfections), but it was uneven across locations (Table 1). We removed 26 infected samples because they lacked negative droplets (n = 21 removed from Marlborough Sounds [*B. ostreae* only = 11, *B. ostreae* coinfections = 8, and *B. exitiosa* coinfections = 2], n = 4 removed from Big Glory Bay [all *B. ostreae* only], and n = 1 removed from Foveaux Strait [all *B. exitiosa* only]).

**Table 1.** Summary table of the 409 sampled ddPCR results across sample location and infection groups. The ddPCR results are from species-specific assays for each parasite. Single-species infections are ddPCR results for oysters infected by either *Bonamia ostreae* or *Bonamia exitiosa*. Coinfections represent ddPCR results for either parasite species, from oysters infected simultaneously by both *B. ostreae* and *B. exitiosa* (all from the Marlborough Sounds). Sampling is uneven between *B. ostreae* and *B. exitiosa* in the coinfected samples because some ddPCR results lacked negative droplets and were excluded

Sample location	Sampled single-species infections (*n*)		Sampled coinfections (*n*)		Location total (N)
	*B. ostreae* only	*B. exitiosa* only	*B. ostreae* from coinfections	*B. exitiosa* from coinfections	
Marlborough Sounds	52	11	91	96	250
Big Glory Bay	53	–	–	–	53
Foveaux Strait	–	106	–	–	106
Total	105	117	91	96	409

**Figure 2.**
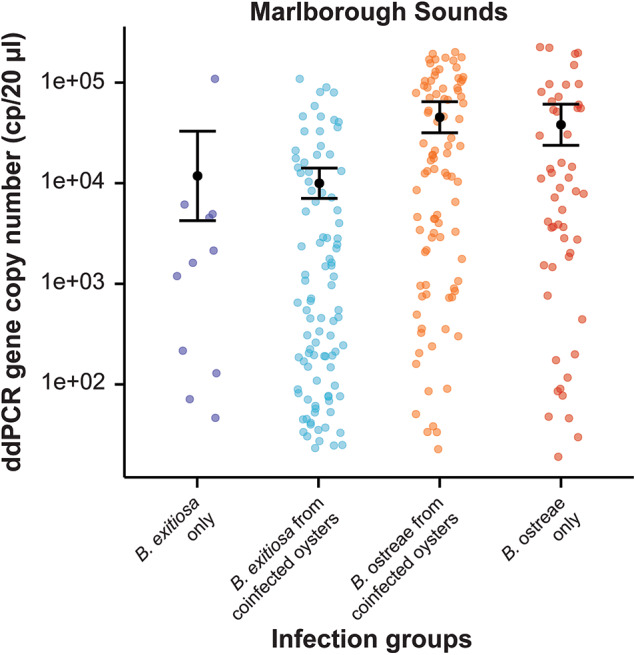
Variation in gene copy numbers (cp/20 µL) for oysters from the Marlborough Sounds, where *Bonamia exitiosa* and *Bonamia ostreae* are sympatric. 18S rRNA gene copies were estimated using the species-specific ddPCR assay for each *Bonamia* species. The sample groups show ddPCR results for single infections of either species, as well as results for each species in coinfected oysters. The black dot represents predicted means and error bars show 95% confidence intervals based on the negative binomial generalised linear model.

**Figure 3.**
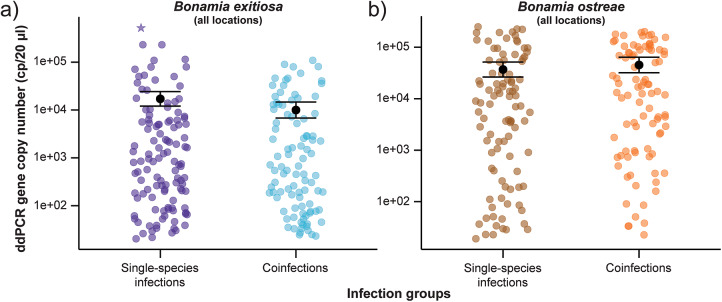
Variation in 18S rRNA gene copy numbers (cp/20 µL) between single and coinfections for (a) *Bonamia exitiosa* (including the influential data point identified by Cook’s distance) and (b) *Bonamia ostreae*, estimated using the species-specific ddPCR assay for each *Bonamia* species. The black dot represents predicted means and error bars show 95% confidence intervals based on the negative binomial generalised linear model. One oyster with a particularly high gene copy number for *Bonamia exitiosa* single-species infections is marked with a star.

**Figure 4.**
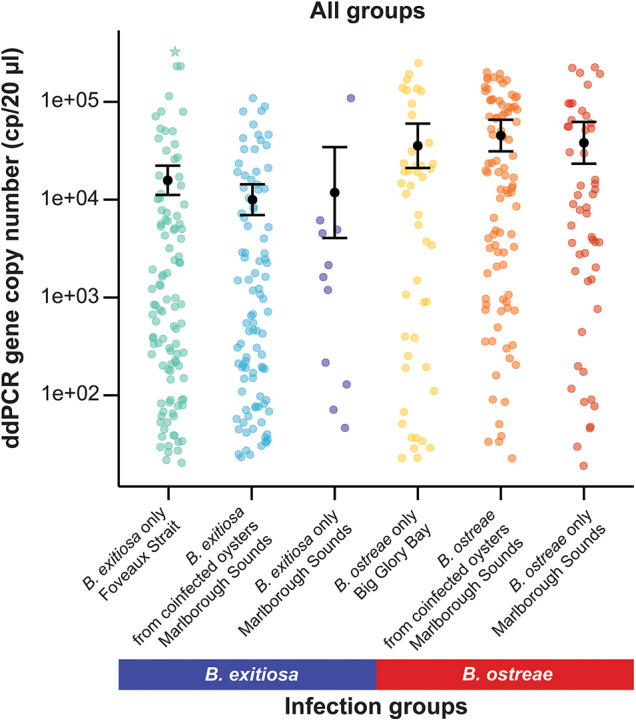
Variation in 18S rRNA gene copy numbers (cp/20 µL) among all tested oyster infection groups and locations. The black dot represents predicted means and error bars show 95% confidence intervals based on the negative binomial generalised linear model. One oyster with a particularly high gene copy number for *B. exitiosa* single-species infections is marked with a star.

The range of *Bonamia* 18S rRNA gene copy numbers for each group spanned orders of magnitude (Figures 2–4). In the Marlborough Sounds where both parasites co-occur, a negative binomial GLM showed higher gene copy numbers in oysters infected with *B. ostreae*, with single infections having a∼ 3·2-fold increase and coinfections a ∼3·8-fold increase relative to *B. exitiosa* (both *P* < 0·001) (Table 2, Figure 2).

**Table 2.** Negative binomial generalized linear model comparing 18S rRNA gene copy numbers between single and coinfections of *Bonamia ostreae* and *Bonamia exitiosa* in flat oysters within their sympatric range in the Marlborough Sounds. The intercept represents *B. exitiosa* single infections and the coefficient for coinfections shows the effect relative to single infections. Estimates are reported on the log scale and exponentiating them gives fold changes relative to *B. exitiosa* single infections

	Estimate	Std. Error	*Z* value	*P* value
Intercept (*B. exitiosa* only)	9.3770	0.5222	17.955	2 × 10^−16^***
*B. exitiosa* from coinfections	−0.1682	0.5514	−0.305	0.7603
*B. ostreae* from coinfections	1.3425	0.5529	2.428	0.0152*
*B. ostreae* only	1.1714	0.5748	2.038	0.0416*

*P* values < 0·05 are shown by

* and *P* values < 0·001 are shown by ***.

For *B. ostreae*, there was no detectable difference in gene copy numbers between single-species infections and coinfections (*P =* 0·4; Supplementary Table S1; Figure 3b). Whereas for *B. exitiosa*, gene copy numbers were 42% lower in coinfections than single infections (*P* = 0·04; Supplementary Table S2). Figure 3a shows one oyster with particularly high copy numbers, which was identified as an influential data point by Cook’s distance (>1·0; Supplementary Fig. S1). Removing it and rerunning the model reduced the estimated difference to ∼21% and the difference between single and coinfections was no longer statistically significant (*P* = 0·3; Supplementary Table S3), indicating that while *B. exitiosa* gene copies in coinfections are lower than in single-species infections, the statistical significance is sensitive to this single oyster.

Across all infection groups and locations, *B. ostreae* consistently showed higher gene copy numbers compared to *B. exitiosa* (Table 3, Figures 2 and 4). For *B. exitiosa*, gene copy numbers from single-species infected and coinfected oysters in the Marlborough Sounds were 33% and 43%, respectively, lower than those from Foveaux Strait (Table 3). The oyster identified as influential in the *B. exitiosa*-only model was less influential in the combined model (Cook’s distance = 0·4; Fig. S2). Despite this, removing that oyster and rerunning the model maintained lower *B. exitiosa* gene copies in Marlborough Sounds, but the difference was no longer statistically significant (Supplementary Table S4).

**Table 3.** Negative binomial generalized linear model comparing 18S rRNA gene copy numbers between all locations and infection groups. The intercept represents *Bonamia exitiosa* single infections from Foveaux Strait and the coefficient for coinfections shows the effect relative to single infections. Estimates are reported on the log scale, exponentiating them gives fold changes relative to *B. exitiosa* Foveaux Strait

	Estimate	Std. error	Z value	*P* value
Intercept (*B. exitiosa* FS^a^)	9.7782	0.1767	55.334	2 × 10^−16^***
*B. exitiosa* from coinfections	−0.5694	0.2563	−2.221	0.0263*
*B. exitiosa* MS^b^	−0.4012	0.5763	−0.696	0.4863
*B. ostreae* BGB^c^	0.6994	0.3212	2.177	0.0295*
*B. ostreae* from coinfections	0.9413	0.2600	3.620	0.0002***
*B. ostreae* MS	0.7702	0.3080	2.500	0.0124*

*P* values < 0·05 are shown by

* and *P* values < 0·001 are shown by ***.

a FS = Foveaux Strait.

b MS = Marlborough Sounds.

c BGB = Big Glory Bay.

## Discussion

### Infection intensity differs between Bonamia species

Gene copy numbers were significantly higher for *B. ostreae* than *B. exitiosa* across both allopatric and sympatric locations, supporting our hypothesis that a recently introduced parasite would produce higher infection intensities than a parasite with a longer evolutionary history with its host. While predicting the effects of invasive parasite species can be difficult, this result aligns with our expectations based on the reported pathogenicity of *B. ostreae* in Europe (Elston et al. 1986) and the observed high mortality in the naive host population following the parasite’s arrival in New Zealand (Lane et al. 2016). While studies of other diseases have reported more severe symptoms in other mixed species infections (de Lima Celeste et al. 2017; Shen et al. 2019; Kotepui et al. 2020; Tang et al. 2020), the overall outcomes for coinfections often seems to depend upon the density and frequency of parasite species (Alizon 2008, 2013; Bolnick et al. 2024). These factors are likely relevant for *B. ostreae* in New Zealand, as it is a recent arrival and presumably less common than *B. exitiosa* at sympatric locations.

*Bonamia ostreae* may cause more severe infections because it has only recently arrived and has not coevolved with New Zealand flat oysters. Since disease is a strong force of natural selection (Carnegie and Burreson 2011), it is almost certain that *B. exitiosa* and *O. chilensis* have exerted coevolutionary pressure on one another. The observed differences in infection intensities of *B. exitiosa* varied between locations, with oysters from Foveaux Strait having higher gene copy numbers than those from Marlborough Sounds (33-43% lower in Marlborough Sounds; Table 4). This difference may reflect host adaptation, as recurrent *B. exitiosa* epizootics in Foveaux Strait could have selected for some tolerance or resistance (Hine 1996; Holbrook et al. 2021). A more tolerant oyster is expected to survive with higher infection loads (Holbrook et al. 2021), which may explain the higher average gene copy numbers observed. While this pattern is consistent with historical epizootics, uncovering the scale and nature of any adaptation of Foveaux Strait flat oysters to *B. exitiosa* is an important line of investigation into disease resilience and oyster management.

The consistently higher infection intensity, as inferred from gene copy number, for *B. ostreae* suggests that disease outcomes are species-driven and that *B. ostreae* will have a negative impact on oyster populations around New Zealand if it continues to spread. Theoretically, the strong selective pressure associated with high infection intensities and high mortality rates for *B. ostreae* infections may cause rapid adaptation among New Zealand flat oyster populations, provided mortality is not so high as to reduce spawner density below levels that would prevent future recruitment. In Europe, a consistent region of genomic variability has been identified for wild and farmed *O. edulis* populations that differ in resistance to *B. ostreae* (Vera et al. 2019; Sambade et al. 2022). Given that *B. ostreae* has spread in Europe since the 1970s (Engelsma et al. 2014), this genomic variation has evolved swiftly within an ecological timescale. Although previous studies have doubted the feasibility of resilience breeding in New Zealand (Ross et al. 2017; Culloty et al. 2020), future researchers could utilise a recent genome assembly (Rodríguez Piccoli 2024) to determine whether a similar genomic region is present and suitable for selection in *O. chilensis*.

### No detectable difference between concurrent and single-species infections

Concurrent infections in sympatric locations produced similar infection intensities to single-species infections, in disagreement with our second hypothesis. Gene copy numbers indicated that *B. exitiosa* and *B. ostreae* have no detectable interactions in concurrent infections. Our ddPCR results are concordant with a previous histopathological analysis, which sampled a subset of the same oysters collected from the Marlborough Sounds in 2015 (Lane 2018). That study found no detectable differences in infection intensity between single-species and concurrent infections (Lane 2018).

The absence of any detectable effect of coinfection compared to single infection on infection intensity is surprising, given the similar life-histories of *B. exitiosa* and *B. ostreae.* Indeed, a well-designed experimental infection could reveal previously unidentified changes in phenotype or tissue tropism of either species during a coinfection. Notably, future considerations need to be given to timing. Samples for this study were collected during fishery or biosecurity operations and therefore represent a sampling snapshot for parasite detection. Individuals with heavier infections could have died before sampling occurred, biasing results towards those individuals with lighter infections. The timing of each oyster’s exposure to *B. exitiosa* and *B. ostreae* during a concurrent infection, as well as the initial parasite load on host contact, are two important points that cannot be controlled given the nature of our dataset. For example, 100% mortality was observed in a mammalian host inoculated with two or more *Plasmodia* species at the same time (Tang et al. 2020), whereas virulence tended to decrease, and specific parasite species were suppressed when inoculated at different times (Tang et al. 2020). It needs to be tested whether similar effects occur with *Bonamia* spp. to provide greater confidence on the lack of interaction observed in this study.

Gene copy numbers remain a proxy of infection intensity and may be an imperfect reflection of biological reality. In lieu of a controlled experiment, however, they do provide a clear indication that there are more copies of the 18S rRNA gene from *B. ostreae* than *B. exitiosa* in an oyster sample. This infection pattern most likely indicates that there are more *B. ostreae* individuals than *B. exitiosa* inside oyster host heart tissue. Alternatively, *B. ostreae* may possess more copies of the 18S rRNA gene in its genome than *B. exitiosa*, which could bias the results towards *B. ostreae*. This hypothesis could be tested via further molecular investigation of both species. Regardless of any variation for the 18S rRNA gene between taxa, the absence of an interaction between *B. exitiosa* and *B. ostreae* in concurrent infections remains unchanged as those estimates depend on intraspecific (rather than interspecific) comparisons.

## Conclusions

Our results show that *B. ostreae* has higher gene copies than *B. exitiosa* in New Zealand flat oysters, suggesting more intense infections, likely reflecting its recent introduction and lack of host-parasite co-adaptation. No significant differences in gene copies were observed between single and concurrent infections, suggesting coinfections do not intensify disease. These findings highlight *B. ostreae* as a continuing threat to *O. chilensis*, while emphasizing the need to assess the long-term impacts of both parasites. By examining congeneric coinfections, this study broadens understanding of *Bonamia* dynamics in New Zealand and underscores the importance of ecological and evolutionary context in marine disease research. Finally, infection intensity insights from gene copy numbers support the value of routine ddPCR screening for high-risk pathogens.

## Supporting information

10.1017/S0031182025100978.sm001 Lane et al. supplementary material 1 Lane et al. supplementary material

10.1017/S0031182025100978.sm002 Lane et al. supplementary material 2 Lane et al. supplementary material

## Supplementary material

The supplementary material for this article can be found at https://doi.org/10.1017/S0031182025100978.
