# Supplementary material for: Concurrent infections by Bonamia species (Haplosporidia) do not cause more intense infections
Source: Parasitology. 2025 Oct 30;153(1):103–10. doi: 10.1017/S0031182025100978 (PMC13215736; doi:10.1017/S0031182025100978)
Supplement: Lane et al. supplementary material 1 — Lane et al. supplementary material [file S0031182025100978sup001.docx]

Supplementary Tables

Table S1. Negative binomial generalized linear model comparing 18S rRNA gene copy numbers between single and coinfections of *Bonamia ostreae* in flat oysters. The intercept represents single infections and the coefficient for coinfections shows the effect relative to single infections. Estimates are reported on the log scale.

|  | Estimate | Std. Error | Z value | *P* value |
| --- | --- | --- | --- | --- |
| Intercept | 10.5158 | 0.1709 | 61.547 | <0.001* |
| *B. ostreae* coinfections | 0.2037 | 0.2462 | 0.827 | 0.408 |

Table S2. Negative binomial generalized linear model comparing 18S rRNA gene copy numbers between single and coinfections of *Bonamia exitiosa* in flat oysters. The intercept represents single infections and the coefficient for coinfections shows the effect relative to single infections. Estimates are reported on the log scale.

|  | Estimate | Std. Error | Z value | *P* value |
| --- | --- | --- | --- | --- |
| Intercept | 9.7466 | 0.1780 | 54.771 | <0.001* |
| *B. exitiosa* coinfections | -0.5378 | 0.2651 | -2.029 | 0.043* |

Table S3. Negative binomial generalized linear model (after removing the influential data point as identified by Cook’s distance) comparing 18S rRNA gene copy numbers between single and coinfections of *Bonamia exitiosa* in flat oysters. The intercept represents single infections and the coefficient for coinfections shows the effect relative to single infections. Estimates are reported on the log scale.

|  | Estimate | Std. Error | Z value | *P* value |
| --- | --- | --- | --- | --- |
| Intercept | 9.4482 | 0.1747 | 54.092 | <0.001* |
| *B. exitiosa* coinfections | -0.2393 | 0.2596 | -0.922 | 0.356 |

Table S4. Negative binomial generalized linear model after removing the influential data point from Foveaux Strait as identified by Cook’s distance. The intercept represents *B. exitiosa* single infections from Foveaux Strait and the coefficients indicate the effect of coinfections, different locations (MS = Marlborough Sounds, BGB = Big Glory Bay), and *B. ostreae* infections relative to the reference. Estimates are reported on the log scale.

|  | Estimate | Std. Error | Z value | *P value* |
| --- | --- | --- | --- | --- |
| Intercept | 9.4553 | 0.1752 | 53.977 | <0.001* |
| *B. exitiosa* from coinfections | -0.2465 | 0.2535 | -0.972 | 0.331 |
| *B. exitiosa* MS | -0.0783 | 0.5689 | -0.138 | 0.891 |
| *B. ostreae* BGB | 1.0223 | 0.3174 | 3.221 | 0.001* |
| *B. ostreae* from coinfections | 1.2642 | 0.2571 | 4.917 | <0.001* |
| *B. ostreae* MS | 1.0931 | 0.3044 | 3.591 | <0.001* |
