# Supplementary material for: Concurrent infections by Bonamia species (Haplosporidia) do not cause more intense infections
Source: Parasitology. 2025 Oct 30;153(1):103–10. doi: 10.1017/S0031182025100978 (PMC13215736; doi:10.1017/S0031182025100978)
Supplement: Lane et al. supplementary material 2 — Lane et al. supplementary material [file S0031182025100978sup002.docx]

Supplementary Figures


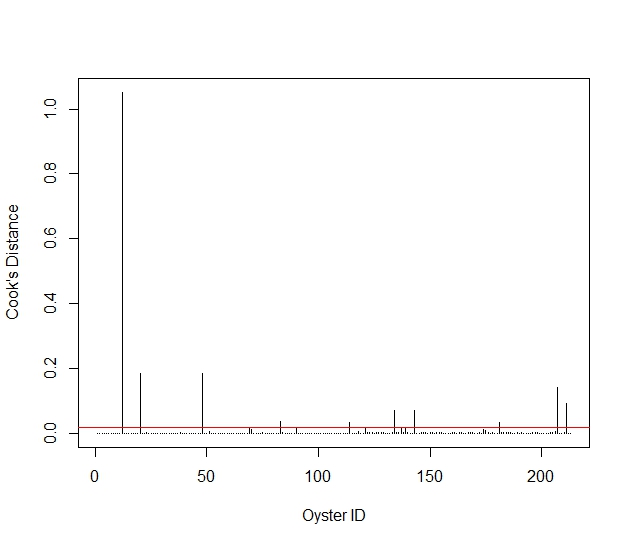


Fig. S1. Influence of individual oysters on the *Bonamia exitiosa*-only negative binomial generalized liner model as measured by Cook’s distance. The y-axis shows Cook’s distance (higher values indicate greater influence) and the x-axis shows oyster ID. Points above the reference threshold (red line) indicate potentially influential data. The oyster with a Cook’s distance >1.0 is from Foveaux Strait.


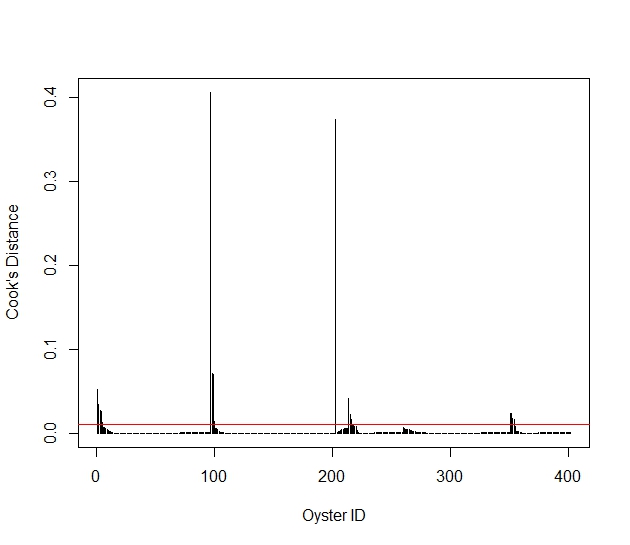


Fig. S2. Influence of individual oysters on the negative binomial generalized liner model for all groups and locations, measured by Cook’s distance. The y-axis shows Cook’s distance (higher values indicate greater influence) and the x-axis shows oyster ID. Points above the reference threshold (red line) indicate potentially influential data. The oyster previously identified as highly influential in Fig. S1 (Foveaux Strait) is oyster #100 in this plot, with a Cook’s distance of 0.4, indicating much lower influence in the combined dataset.
